# Ethical Perspectives of Therapeutic Human Genome Editing From Multiple and Diverse Viewpoints: A Scoping Review

**DOI:** 10.7759/cureus.31927

**Published:** 2022-11-27

**Authors:** Andrew M Joseph, Monica Karas, Yaseen Ramadan, Ernesto Joubran, Robin J Jacobs

**Affiliations:** 1 Osteopathic Medicine, Nova Southeastern University Dr. Kiran C. Patel College of Osteopathic Medicine, Fort Lauderdale, USA; 2 Research/Health Informatics/Medical Education, Nova Southeastern University Dr. Kiran C. Patel College of Osteopathic Medicine, Fort Lauderdale, USA

**Keywords:** public perspectives, genomic editing, human fetus, theology, philosophy, genomics ethics, genomic engineering, genetic engineering

## Abstract

Human genome editing has been increasingly explored to determine if it can be used to eradicate genetic diseases like sickle cell disease, but it has also been surrounded by a wide variety of ethical dilemmas. The purpose of this review was to conduct a scoping review of the ethics of therapeutic human genome editing in terms of philosophy, theology, public perspectives, and research ethics. A systemized search of PubMed, Embase, Ovid MEDLINE, and Web of Science was conducted. The initial search resulted in 4,445 articles, and after removing 1,750 duplicates and screening the remaining 2,695 articles, 27 final articles were selected for the final analysis. From a philosophical and theological standpoint, therapeutic human genome editing was generally ethically acceptable. Worldwide public perspectives were also in agreement except for the Oceanic region, which disagreed mainly due to the possible effects on future generations. Lastly, human research ethics revealed that women were not always included in informed consent, and that child autonomy needs to be preserved. Further research is needed to determine adverse effects on the mother, fetus, and future generations.

## Introduction and background

Genetic engineering has also been used to modify viruses to vaccinate against specific diseases, which has increased awareness of the various possible applications of genetic engineering [1]. The news of the genetically edited twins in China in 2018 specifically brought to question the ethics of therapeutic gene editing in human embryos [2]. Although there are no current regulations in place for human genome editing, the topic has been widely debated globally in various domains [3]. The purpose of this study is to conduct a scoping review of the ethics of human genome editing in terms of philosophy, theology, public perspectives, and research ethics.

Human genomic engineering

Genomic editing has become a technological phenomenon that arrived at the forefront of future medical treatment, even in utero. Work in the area of genomic editing began in 1970 with Paul Berg, who developed technology on the use of recombinant DNA in the alteration of other living organisms [4]. These advancements allowed scientists to begin using these technologies to alter an organism's DNA in order to create certain products, in the case of bacteria, or enzymes, in the case of plants or yeast [4]. Initially, such editing was limited to specific organisms, such as mice or yeast, but as the technology progressed, it began to allow for use of the technology on humans, changing the course of treatment in the medical field [4].

The beginning of human genomic editing lay with two gene-editing techniques that relied on the use of restriction enzymes in editing genomes: zinc finger nucleases (ZFNs) and transcription activator-like effector nucleases (TALENs) [5]. ZFNs have a zinc finger DNA binding domain that is used to bind a specific target DNA sequence as well as a restriction endonuclease domain used to cleave the DNA at the target site. TALENs are also composed of DNA-binding domain and restriction domains like ZFN, but their DNA-binding domain has more potential target sequences than the ZFN gene-editing tool allows to use [5]. The use of these technologies predominated the field for years, but their difficulty, expenses, and time-consuming nature made further research into the field difficult [5]. However, in 2012, it was discovered that *Streptococcus pyogenes*, a bacterium, utilized a system for defense against viral infection that could be outfitted for use as a system for genomic editing [6]. This system consists of two parts: the first part is the “clustered regularly interspaced short palindromic repeat” (CRISPR) of RNA that can be used to act as a guide for genome targeting, and the second is the “CRISPR-associated protein 9” (Cas9) that acts as an endonuclease which enables the use of double-stranded breaks of the DNA [6]. As such, it was possible for scientists to be able to manipulate the CRISPR RNA molecule into a single guide RNA (sgRNA) that could be engineered to specifically target a genomic area of interest in humans [6]. This technology was soon updated and adapted to be able to edit the human genome with much higher efficiency and selectivity than before [6].

The overall application of gene-editing tools varies, and their use depends on the cell lines and organism involved along with whether the final goal is clinical or research [7]. These types of editing, in particular CRISPR-Cas9, allow scientists to target multiple loci of DNA in a single human being and allows for human genome-wide screening and alteration [7]. Therefore, scientists are looking in the field for further implications and applications of these technologies. It was found that CRISPR provides a simple tool that can point to any sequence of a genome just by designing a simple sgRNA sequence [7]. Even in the past few years, CRISPR has gone even beyond altering just DNA, it has seen use in altering chromatin and targeting epigenetic regulation [7]. CRISPR has become so precise that it can now achieve large-scale functional screenings of the genome by using guided proteins with thousands of copies of sgRNA in each targeted cell in order to recognize genes that are known to code for an explicit phenotype in a human being [7]. Applications of CRISPR vary, including being able to alter defective genes, such as the beta-globin gene in sickle cell anemia in order to achieve therapeutic levels of normal red blood cells [8]. CRISPR can also be looked at to alter oncogenic genes in order to prevent cancer from developing; one example is its use in restoring p53 in the carcinogenesis of lung carcinoma [8]. Another possible use of CRISPR has been seen in the process of reducing telomerase activity in aging or cancer [8]. CRISPR has been explored for many various immunological and genetic diseases for possible therapeutic options with much success, although as the limits begin to disappear for this technology, a question has begun to arise of how far it should be taken, and whether we have gone past the point of beneficence in treatment.

The role of ethics

Ethics is a salient component in the field of medicine. One main ethical issue is the determination of human nature. It is arguable that any genetic interference that could change human nature should be morally forbidden since it alters the very essence of human nature [9]. This practice would also run the risk of designating humans to a predetermined life, which consequently adds restrictions on freedom of choice [9]. The prospect of performing germline alterations on living creatures targets the field of medicine by decreasing the genetic pool. This can produce negative repercussions: diminution in heterozygosity and uniformization of genes involved in recombination. Moreover, germline interventions might not be reversed or altered, whenever need demands it. This argument is questioned by safety, population versus individual focus, spontaneous mutations, exceptionalism, the intentional pursuit of genetic diversity through germline interventions, and harm reduction potential [10].

There are several types of ethics, some being cultural, philosophical, research-centered, and theological. For example, western nations hold less sympathetic views of changes in embryos compared with somatic cell edits than non-western nations [11]. Another division of moral philosophy is normative ethics (i.e., what should be done), while applied ethics tackles practical themes, such as war and capital punishment. Ethics in medical research deals with the conflicts of interest between healthcare entities, care providers, and patients, such as autonomy, non-maleficence, beneficence, and justice [11]. Finally, from a theological perspective, helping humankind is the basic goal of science and is part of God’s desire for cooperation with mankind. Caution must be taken when reaching limits that might be unethical [12].

Ethics and human genomic editing

Ethics ultimately provides the basis for either justifying or discrediting scientific advancements, and gene editing also must be scrutinized under the same microscope [13]. While there are a tremendous number of applications pertaining to genomic editing, there are many purposes that should seriously be discussed from both a social and moral perspective [14]. The genesis of new, simplistic, and easy-to-use applications for human genomic editing, such as CRISPR-Cas9, TALENs, CAR T-cells, and others opened the door for an even wider range of applications [13]. Prior to 2015, any utilization of genomic editing for humans was reserved exclusively for somatic cell lines [14]. However, CRISPR-Cas9 made it possible to modify the germline of humans for the first time [14]. With this new generation of gene editing modalities, disorders such as sickle cell disease, primary immunodeficiency, retinal or dermatologic disorders, and many others can be managed and potentially treated [15]. Despite the endless possibilities of advancements that the new generation of gene editing modalities brings, new bioethical, moral, and social-ethical issues have emerged that may provide a warning regarding their utilization [14].

Human genetic modification is not new to the ethical debate spotlight [13]. As with other facets of science, there must be a balance between the benefits and risks [15]. These new human gene editing techniques have not been perfected and as such need to be carefully analyzed [16,17]. The question beckons - how does one identify its efficaciousness if it is not in mainstay practice [18]? To answer this question, therapeutic legitimacy must be identified starting in individual cases [18]. Informed consent and self-determined consent of the patient are often the cruces of legitimizing a treatment protocol [18]. However, if it is to be used on an embryo, then the paradigm shifts from therapeutic legitimacy to therapeutic benefit [18]. It is critical to differentiate between preventative and therapeutic measures as this can misrepresent the true application of a management plan [18].

Overall, the current literature regarding the ethics of human genomic editing boils down to the following four distinguished themes - philosophy, theology, cultural and public perspectives, and overall ethics in research. From a philosophical perspective, the primary goal of biomedical advancements is to improve the human condition [19]. Whether this means pushing the boundaries and limitations of mankind or fostering the improvement of the “current version” of humans, it must be done with the utmost respect and delicate precision [19]. From the theological perspective, secular and religious bioethicists have described genomic editing and its applications as “playing God” [20]. In various religions, such as Christianity and Islam, mankind was made in the image of God; therefore, the question has always been if humans are called to play God or not [20]. The vast array of cultural and public perspectives on genomic editing can be attributed to many factors [21]. Such factors that split perspectives include nationality, religion, political affiliation, and the disorder or disease being addressed [21].

Despite all the literature that is currently on the topic of ethics and genomic editing, no research has reviewed the current state of the science regarding the new generation of therapeutic human genomic editing modalities such as CRISPR-Cas9, TALEN, CAR T-cells, and the like with respect to each major theme identified. Therefore, the purpose of this scoping review was to map out the literature on the ethics of therapeutic human genome editing regarding philosophy, theology, public perspective, and research ethics.

## Review

A computerized search using the databases Embase, Ovid MEDLINE, and Web of Science was conducted to map out the published literature on ethical perspectives and implications in the field of genetic engineering.

Search strategy and identification of studies

The inclusion and exclusion criteria were established prior to conducting the review. Articles were included if they were in the English language, published between 2013 and 2022, analyzed humans only, and included the keywords “genetic engineering,” “genomic engineering,” and “ethics.” Studies that did not include primary data were excluded (e.g., book chapters, editorials, newspapers, print media, book reviews, conferences, abstracts, and erratum). Using Boolean operators, the search terms were used in all three databases. The detailed search queries per database can be found in the Appendix.

Data extraction

**Figure 1:**
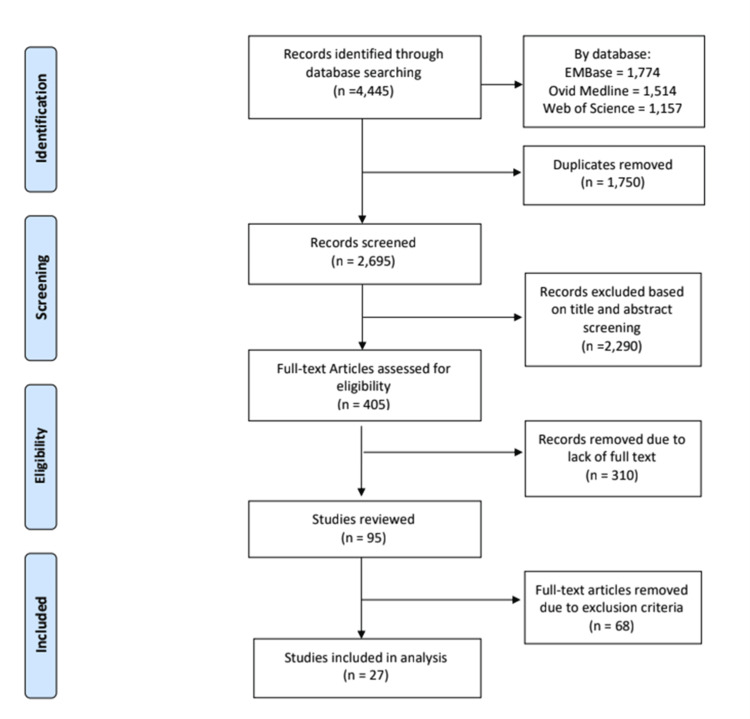
PRISMA flow diagram. PRISMA: Preferred Reporting Items for Systematic Reviews and Meta-Analyses

Using the search criteria, the initial search yielded 4,445 articles. After removing 1,750 duplicates the remaining 2,695 were further screened for inclusion criteria. Articles in the format of a paper, book review, correspondence, editorial, print media, erratum, or conference abstract were excluded (n=405). The remaining 2,290 articles were then removed due to the abstract not including all the keywords “genetic engineering” or “genetic editing” or “genomic editing” and “ethics.” Next, the remaining 405 articles were assessed for the availability of access to full text, and 310 articles were removed. The inclusion and exclusion criteria were then used to assess the remaining 95 full-text articles; a total of 27 articles were selected for final analysis (Figure 1).

Results

Table 1 reports the characteristics of the 27 primary studies included in the review whereby the factors and ethical along with cultural, occupational, and geographical background considerations were considered. The studies on therapeutic human genomic editing were grouped into the following four major domains: philosophy, theology, public perspectives, and research ethics.

**Table 1:** Summary table of articles used for the review. GGE: germline genetic engineering; MENA: Middle East and North Africa; HGE: Human Genome Editing

Study	Study description/purpose	Sample	Major findings and limitations
Philosophy			
Morar (2015) [9]	To assess the validity of Habermas’ argument against genetic enhancement.	N/A	The author argued against Habermas’ argument, claiming that it was a series of assumptions that mischaracterized evolution and that the facts presented did not have data to support them.
Segers and Mertes (2020) [22]	To explore how human dignity is invoked normatively in relation to heritable genome editing.	N/A	Human germline gene editing has the potential to reinforce and violate human dignity. This means that considerations about human dignity should be included in the calls for debate over the ethics of genome editing, but there is no need to come to a complete standstill.
Chan (2015) [23]	To discuss how international guidelines of ethics utilize the concept of human dignity and its application to restricting genetic engineering.	N/A	The issue with utilizing human dignity as a point of debating the use of genetic engineering is that different people interpret the concept differently resulting in different opinions as to the acceptability of engineering. Thus, clarifying that dignity has different meanings may help to create a better understanding of the debate. He states that there is a hidden assumption in debates about the role of dignity in guidelines on bioethics, which may help to recognize what is at issue between people who disagree about the forms of genetic research and technology that are morally acceptable.
Raposo (2019) [24]	To address and rebuke the argument that gene editing violates human dignity and is not compatible with human nature.	N/A	Human dignity won’t be an obstacle to gene editing once an understanding of human dignity is achieved. Then, justice will be done to its rich philosophical background and simultaneously will be able to meet current needs. Such understanding of human dignity must be one that expresses a characteristic that inherently differentiates the human person from other creatures: the power to decide destiny and to develop in order to become the best version of oneself. Thus, human dignity is respect for human autonomy.
Alonso and Savulescu (2021) [25]	Analyze He Jiankui’s case in relation to one of the most difficult problems in procreative ethics: the non-identity problem. The analysis will help to understand the ethics involved in gene editing and hopefully allow for better, more philosophically grounded legislation on CRISPR and other gene-editing technologies.	N/A	It is a slippery slope to decry the birth of these twins because it seems incorrect to say they should not have been born, but to accept what was done is a slippery slope to further irresponsible genetic modifications.
Li and Zhang (2019) [26]	To analyze all the ethical arguments against genomic editing and point out their merits and flaws. It then juxtaposes a Confucianism approach to find a new perspective to determine whether designing babies with CRISPR is ethical.	N/A	As of now, both sides to the argument have been unable to completely disprove the other. A very specific argument by Sandel vehemently goes against embryo gene designing (EGD). On the one hand, Confucianism is able to address many questions unanswered by Sandel’s argument, such as who gives children to parents as gifts. On the other hand, unlike Sandel’s total denial of EGD, Confucianism holds a context-sensitive attitude towards EGD. That is, Confucianism would allow EGD if it benefits a family’s prosperity and integrity. The main points against EGD are future generations deciding, playing God/nature, commodifying, and prejudice. The main points for EGD are liberty, well-being, and risk.
Gyngell et al. (2017) [27]	To analyze the ethical arguments for and against pursuing germline genetic engineering and the effectiveness of these arguments.	N/A	Calls for bans on GGE should be resisted as there are more medical benefits than possible risks for the use of it. While issues like future consent, enhancement, and safety are at the forefront of the argument against GGE, the medical benefit of disease eradication and the act of being cautious mitigate these risks.
Theology			
Alsomali and Hussein (2021) [28]	To argue that from an Islamic standpoint, the therapeutic application of CRISPR-Cas9 for germline editing may be permissible if the safety and efficacy concerns are resolved and if the principles of Maqasid al-Shari'a are fulfilled.	N/A	(1) Decisions from an Islamic perspective rely on the application of Maqasid al-Shari'a and Qawaid Fiqhiyyah as the sources of ethical guidelines for the evaluation of novel technologies, including CRISPR-Cas9 from the Islamic bioethics' perspective. (2) Multi-disciplinary experts, including geneticists, Shari'a law specialists, bioethicists, and social scientists, will need to work together to generate appropriate ethical, religious, and moral conclusions regarding the use of CRISPR-Cas9 in the Muslim world. (3) CRISPR-Cas9 may be permissible for therapeutic applications, including germline editing, based on necessity, once concerns regarding safety and efficiency have been resolved.
Isa et al. (2020) [29]	To discuss the principles of preservation of human life, lineage, and dignity and the fact that preventing harm takes precedence over securing benefit are among the guiding principles in assessing the permissibility of CRISPR-Cas9-mediated human germline editing, within an Islamic perspective.	N/A	Based on this study, it can be concluded that CRISPR-Cas9-mediated human germline gene editing would be considered lawful in Islam if it met the following conditions: (a) it is only used for medical purposes particularly to prevent or treat diseases. Such a modification is not considered tampering with God’s creation. (b) It is allowed only after safety and efficacy issues are resolved. The technology used should not bring more harm to the parents, the resulting child, society, and the future generation. (c) Strict regulation is established to ensure respect for the persons involved, prevent premature use, and abuse of the technology as well as strictly prevent human genetic enhancement.
Loike and Kadish (2018) [30]	To analyze, from a Jewish legal perspective, some of the ethical conundrums that society faces in pushing the outer limits in researching these new biotechnologies.	N/A	The general rule in Judaism is that gene editing for non-medical applications is ethically wrong and should not be routinely acceptable. The Torah states that its laws are created for people to live by, and so medical and technological advances that promote the saving of lives should be supported. In the realm of new biotechnology, the goal of partnering with God to save lives should be paramount.
Peters (2017) [31]	To assess how CRISPR-Cas9, like so many other new biotechnologies, is forcing a moral choice on a large scale. Gene editing for purposes of medical therapy, human enhancement, engineering future children, and even creating a posthuman species, confronts our society with the inescapable necessity of making moral choices.	N/A	Human creativity belongs to God and should be morally guided even in self-transformation, and not suppressed. Human’s relationship to self, God, and the world should be taken into consideration. Using CRISPR-Cas9 to improve human health by advancing medical technology would not violate the image of God. Therefore, therapeutic somatic gene editing is ethically acceptable. However, although gene editing for enhancement purposes brings up ethical questions, it does not threaten human nature, and therefore requires further discussion. Since human germline editing has further consequences for generations to come, further discussion and research are required to determine future practical applications without violating ethical and moral standards.
Peters (2019) [32]	To explain the split between scientists with regards to the extent of genetic engineering and how much of a backlash from nature could be expected from using this technology and provide an opinion as to the validity of this perspective.	N/A	First, encourage the scientific community to remain in the conversation regarding bioethics without separating the professions. Second, bioethical research projects should be funded to examine the long-range impact of germline modification via CRISPR or other genome modification methods. Overall, a more cautionary approach should be taken towards eugenics, a balance between flying forward towards a posthuman future and stopping all advancements for a boogeyman that may not exist.
Public perspectives			
Rubeis and Steger (2018) [33]	To analyze the ethical implications, risks, and benefits of genome editing.	N/A	The trajectory of the genome editing techniques suggests that it could one day be safe. Society’s perspective of its risk could be appeased by the presence of regulation.
Vasquez-Loarte et al. (2020) [34]	To identify patients’ beliefs and values of gene editing as a therapy for hemophilia in themselves and their relatives.	Contacted 21 individuals in the United States who either had hemophilia A/B or were a parent of someone with it.	Gene editing was not immediately accepted due to an insufficient amount of research and clinical data along with hesitancy to manipulate the genetics of the unborn baby. Data were inclined to favor such therapy if more research was done.
Howell et al. (2022) [35]	To assess perceptions of the risks and benefits of types of gene editing in the United States.	An online survey was completed by 1,600 adults from the United States that addressed perspectives on heritable edits for enhancement, heritable edits for therapy, non-heritable edits for enhancement, and non-heritable edits for therapy.	Revealing therapeutic benefits information yielded more positive views and support for human gene editing. Revealing heritable edits information yielded more negative views about and less support for human gene editing. No difference between risk and benefit perspectives of heritable and non-heritable edits.
Shozi (2021) [36]	To analyze how one African perspective may justify the application of human germline genome editing.	N/A	According to a particular African perspective called “Ubuntu,” the grounds for using human genome editing applications are contingent on whether the autonomy of the child is maintained or not. Therefore, there is no justification for an entire inhibition of germline genome editing.
Ebeling and Gebhard (2022) [37]	To assess the perspective of German young adults on genome editing and its impact on society and nature.	Forty 20-24-year-old students and 57 16-18 years old students	Based on the audio recordings obtained in the group discussions, only negative fantasies and myths were articulated regarding gene editing.
Watanabe et al. (2020) [38]	To assess how the media in Japan covered genome editing affected the public opinion of genome editing.	Japanese people ranging from the age 20-60 years.	Respondents to the surveys were overall approving of genome editing when exposed to medical applications of the technology. Respondents to the surveys were overall opposed to genome editing when exposed to the news about its use with human fertilized eggs.
Hudson et al. (2019) [39]	To explore the spectrum of Māori perspectives on the risks and benefits of gene editing.	Eight key Māori informants.	Māori informants were not overall against new gene editing technologies; however, desired a dynamic approach based on the specific case of use.
Hendriks et al. (2018) [40]	To assess the general Dutch opinions on gene modification.	1,013 Dutch individuals ranging from 11 to 90 years old.	Subjects who were younger, male, or had seen the documentary on gene editing implications were more likely to accept its use in more cases.
Abuhammad et al. (2021) [41]	To identify any ethical challenges that the MENA region would encounter with the introduction of gene editing	28 researchers from the Middle East and North Africa region	The researchers shared the belief that gene editing for treating genetic conditions was important; however, suggested the presence of regulation to ensure no misuse of the technology.
Hollister et al. (2019) [42]	This study investigates the views of the sickle cell disease (SCD) community. We utilized a mixed-methods approach to examine SCD stakeholders' views in the United States.	N/A	SCD holders, while equally worried, are more enthusiastic about and more likely to use HGE than the public if it provides a much-reduced risk of serious diseases for their child. Significantly more SCD stakeholders indicated that they probably or definitely would use HGE to give their baby a much-reduced risk of serious diseases or conditions over his/her lifetime when compared to the general population from Pew (v2=13.92, p=0.0002) and African Americans from Pew (v2=21.33, p<0.0001). This pattern was consistent when participants were asked about how enthusiastic they were about the possibility of HGE for society as a whole. SCD stakeholders were more enthusiastic than the general Pew population (v2=27.21, p<0.0001) and Pew African Americans (v2‡38.67, p<0.0001). However, SCD stakeholders were equally as worried about the possibility of HGE for society when compared to both the general Pew population (v2=0.05, p=0.82) and Pew African Americans (v2=0.11, p=0.73). As the large majority of SCD stakeholders in our sample identified as African American, we felt it was appropriate to compare their opinions to both groups.
Petre (2017) [10]	To investigate the morality of human germline editing from the perspective of future generations.	N/A	From the future generation’s perspective, editing could lead to the reduction of heterozygosity, which is correlated with a health or performance advantage and the uniformization of the genes involved in reproductive recombination, which may lead to health risks when it comes to asexual reproduction. As such, germline interventions aimed at modifying the genomes of future people cannot be ethically justifiable if there is no possibility of controlling the intervention either by reversing or altering it.
Research ethics			
Getz and Dellaire (2020) [43]	The study examines Dr. He’s principles under the perspective of Beauchamp and Childress’ Principles of Biomedical Ethics, as well as that the “clinical future” of heritable genome editing was made clear on the basis of Dr. He’s proposal.	N/A	Five principles are examined in regard to gene editing: mercy for families in need, only for serious disease-never vanity, respect a child’s autonomy, genes do not define you, and everyone deserves freedom from genetic disease. Each of these five principles presents limitations which leads to the conclusion that human gene editing performed for medical purposes presents no basic moral dilemmas, while human gene editing performed with the goal of enhancing an individual defies ethical principles.
Malmqvist (2021) [44]	This study examines the event of two twin girls born in China after being genetically edited.	Participants in potential experimentation could be individuals who do not desire a healthy genetic child at all costs or that can fulfill their desire for offspring by other methods, adoption for example.	It has been concluded that an acceptable methodology for gene editing is hardly uncovered; this represents an obstacle to human germline editing. This paper argues that allowing this procedure to be the norm would conflict with the research ethic principle of non-exploitation.
Zang and Yueqin (2021) [45]	This study takes into account both benefits and risks of human gene editing. It shows that different applications depending on their purposes may be justifiable or not.	N/A	Thanks to progress in science, alteration of human characteristics is now possible; however, gene-editing technology is confronted by limits of ethical principles. For this reason, several experiments were banned in previous years.
Farrell et al. (2019) [46]	This article ought to explore the perspective of women as patients in gene editing, along with its repercussions on women's health and well-being.	Volunteering women free from any type of coercion.	This article explains why excluding women from the discussion of germline editing is unjust as well as the importance of informed consent. Women must have priority in the decision to use gene editing. Limitations however are represented by the fact that innovation needs researchers, ethicists, policymakers, and other key stakeholders to dynamically cooperate with women.

Philosophy

Articles discussing ethics in therapeutic human genomic engineering through a philosophical perspective argued using Habermas’ argument, human dignity, the non-identity problem, and the Confucianism approach. Morar argued against Habermas’ argument, supporting genomic engineering [9]. Segers and Mertes and Chan argued that genomic engineering can both violate and reinforce human dignity, while Raposo posited that human dignity is not violated in genetic modification [22-24]. Petre discussed using the future generations’ perspective that genetic modification may lead to the uniformization of genes involved in reproductive recombination, leading to health risks [10]. Alonso and Savulescu analyzed He Jiankui’Ys gene-editing experiment on the Chinese twins and the ethical dilemma surrounding the experiment by discussing the non-identity problem [25]. Li and Zhang analyzed the ethics of embryo gene designing (EGD) by using the Confucianism approach and concluded that this approach allows therapeutic EGD with risks including prejudice, playing God/nature, and commodifying [26]. Finally, Gyngell et al. analyzed the arguments surrounding germline genetic engineering (GGE) and concluded that GGE should not be banned since the medical benefits of disease eradication outweigh the risks [27].

Theology

The field of theology surrounding the ethics of genomic engineering revolved around the principles of Islam, Christianity, and Judaism. Alsomali and Hussein and Isa et al. both concluded that therapeutic applications of human genomic modification are ethically justified from an Islamic perspective, but strict regulation needs to be put in place by multi-disciplinary experts [28,29]. Peters and Peters also argued similarly through a Christian perspective [31,32]. Loike and Kadish also justified the use of therapeutic human genomic editing through a Jewish perspective and added that non-medical applications are ethically unjustified [30].

Public perspectives

In this section, articles discussing the ethics of genomic editing were divided based on the culture, geographical location, and occupation of the sample population being studied. Rubeis and Steger analyzed the ethical implications worldwide and concluded that although genomic engineering can be done safely one day, society’s perspective can be appeased by the presence of regulation [33]. Vasquez-Loarte et al. analyzed the perspective of patients with hemophilia and concluded that more research needs to be done for patients to favor such therapy due to the uncertainty surrounding it [34]. In the US, Howell et al. conducted an online survey of 1,600 adults and revealed that people view the therapeutic benefits positively, but do not like the risk of heritable human genomic editing that can be passed down to future generations [35]. Shozi analyzed the argument centered around human dignity from an African perspective and concluded that the Ubuntu perspective supports therapeutic human genomic editing only if the child’s autonomy is maintained [36]. German young adults surveyed by Ebeling and Gebhard in group discussions revealed that only negative myths were discussed regarding gene editing [37]. The impact of the Chinese twin babies in Japan resulted in overall support of therapeutic human genomic editing, but disagreement with germline gene editing with human fertilized eggs [38]. Māori indigenous perspectives of New Zealand revealed that the eight informants were not entirely against genomic modification, but required a dynamic approach based on the application [39]. A survey of the Dutch population concluded that younger males or people who have previously watched a documentary on human gene editing were more likely to accept the therapeutic applications of human genomic engineering [40]. Studies from the Middle East and North Africa (MENA) region and sickle cell disease stakeholders were in favor of therapeutic human genomic editing but recommended regulations to be in place to prevent misuse and consider risks to future generations [42-44].

Research ethics

Regarding research ethics, articles discussed the ethics pertaining to research in therapeutic human genomic engineering. Getz and Dellaire identified five principles that are supported by therapeutic human gene editing but are clearly violated by genetic enhancement purposes [43]. Farrell et al. also discuss the ethical principles that prevented several experiments on human genomic engineering from being conducted [46]. Malmqvist analyzed the research ethics portion of the Chinese twin babies edited by Dr. He and argued that allowing this experiment to be the norm would conflict with the research ethics principle of non-exploitation [44]. Lastly, Farrell et al. analyzed the perspective of women as patients in human gene editing and emphasized the importance of informed consent of women participants in clinical trials of genomic editing [46].

Discussion

Philosophy

The philosophical differences that come with therapeutic human genetic engineering are vast, and the various principles must be considered before genomics can be universally instituted. One recurring theme is dignity, which seems to be violated directly by human genomic engineering [22]. While a complete shutdown of genetic engineering is not justifiable, careful considerations must be made with its use [22]. Many believe that if dignity can be understood properly and combined with our current needs as humans, therapeutic genomic editing can assist in achieving the best version of ourselves [24]. An inherent limitation to using dignity as a cornerstone of therapeutic human genetic editing is its variability in interpretation from person-to-person [23].

At the forefront of any discussion about the ethicality of therapeutic human genomic engineering is the work of Dr. He Jiankui, the scientist who genetically engineered two children using CRISPR to make them immune to HIV [25]. His experiment, when revealed, kicked up a storm of controversy and set forward a discussion of whether it was philosophically and ethically correct or not [25]. From a philosophical standpoint, a non-identity problem arose. This means that, despite any morally good or bad actions, it can be reconciled by the fact that those involved did not have their lives changed for the better or worse [25]. However, the potential altering of life makes this act questionable, and the community was stuck between not wanting to decry the births of these children, and still having to condemn the act to prevent a slippery slope for further unregulated editing [25]. Habermas, another famous figure in the argument against human gene editing, argues against editing on the grounds that it went against human nature and should be considered as cheating evolution [9]. However, many have found Habermas’ arguments as misguided and with very little data to support such claims [9].

Despite all the arguments that may arise against human genetic editing, there are many who argue that any philosophical issue is outweighed by the medical benefits [27]. Although future consent, concern about enhancement, and safety are important issues that must be considered, the possibility of disease eradication is a very strong argument in favor of genome editing, particularly in the setting that there has not been any definitive proof of such drawbacks yet [27]. As of now, neither side has been able to provide a solid evidence-based reason to disprove the other’s perspective; therefore, the technology proceeds with caution. While taking issues like prejudice and parity into consideration is important, the advantages in liberty and well-being conferred by technologies like CRISPR provide a strong argument to continue working towards developing the idea of gene editing further [26].

Theology

A theological perspective was perhaps one of the first arguments to develop in relation to human genetic engineering. For example, Islam is shown to take a similar approach to the typical cautions and indications of human genetic editing, allowing it only when any safety or efficacy issues have been resolved [28]. However, there is an added stipulation that a multi-disciplinary team of Sharia law specialists comes together with bioethicists and scientists to work out the appropriate ethical, religious, and moral conclusions of human genetic editing [28]. The consensus is that it is permissible when used for medical purposes in the treatment of disease, as such modifications are considered religiously acceptable and within the frame of God’s vision of us [29]. From a Jewish perspective, genetic editing, when used for medical reasons, can be seen as a collaboration between humans and God to be able to save lives as the Torah and its laws were always made for people to live by [30].

Generally, Christian theology takes a more nuanced approach. Human creativity is at the forefront of if genomic editing is acceptable [31]. The human creativity that comes with advancing technology like human genetic editing is inherent to God, as humans are created by God who always aims to do new things [31]. Thus, any technology guided by human creativity should be encouraged, not stopped, so long as it is done ethically with respect to the relationship with God and the world [31]. The major implication then is that if therapeutic genome modification has the potential for improving human health, then the divine image of God that is present in every human will lead to the use of CRISPR’s benefits [31,33].

Overall, all the different religions and theologies have a similar perspective, with some iteration of allowing the use of human genomic editing if it is done medically or therapeutically and within what is considered “God’s image.” However, similarly to the practical perspective, there is a consensus across all religions that more research and meetings between scientists and religious experts are needed to discuss the technicalities and smaller aspects of what is appropriate per religion. The limitations of these perspectives are that while there are common threads throughout each theology, there will always be subtle differences between each religion.

Public Perspectives

As with other scientific advancements and discoveries, human genome editing is the latest to be scrutinized for potential therapeutic applications and assessed for potential risks and benefits [33]. From a medical standpoint, human genome editing could usher in a new wave of disease treatment and management; however, from an ethical point of view, it remains to be seen [33]. The public’s perception of the risks and benefits has shown to be more of a spectrum of perspectives that depends on the region, the scenario of its use, and the ethical implications [33,34].

In the United States, two studies were identified that assessed the ethical beliefs and values of therapeutic genome editing for hemophilia and for overall use [34,36]. In the study regarding its use for hemophilia, 21 participants from different states, who either had or were related to someone with severe disease, were interviewed to assess if new human genome editing modalities should be considered as a treatment modality [34]. Overall, if genome editing was found to have mild adverse effects on the mom or fetus, then it would be viewed as a possible acceptable treatment [34]. Ethically, the biggest concern was the cascade effect on the unborn offspring [34]. The consensus was that the mainstay treatment is preferred as it is more established from both a safety and efficacious standpoint [34]. Expanding to more general uses, one study sent a survey to 1,600 United States adults to assess the perspective for therapeutic versus enhancement use and whether it was for heritable versus non-heritable applications [35]. Irrespective of if genome editing would be used for heritable or non-heritable applications, the majority had endorsed it with favor if it were strictly therapeutic in nature [35].

In Europe, two studies were identified that evaluated public opinion on human genome editing from an ethical perspective [38,41]. In the Dutch study, a survey was completed of 1,103 participants to determine the acceptability of different uses of human genome editing [40]. Applications ranged from curing diseases versus enhancement to curing genetic diseases versus preventable diseases [40]. While Hendriks et al. identified these perspectives, they noted that the reasons for these findings were predicated on if the treatment management had outweighed the potential risk and ethical concerns present with genomic editing [40]. Ultimately, this study suggested that there is an initial acceptance of genomic editing only if it is used for non-enhancement and pathology that does not already have an equivalent management or treatment modality [40]. The German study utilized generally accepted myths focused on human genome editing [37]. Such myths included how it could be used to “widen the gap between poor and rich,” how it could pollute the “biogenetic parent-child relationship,” and that its use could “lead to humans losing their natural status” [37]. These findings, when interviewing 97 German adolescents with “good levels of education,” suggest that there is a perceived association in adolescents between human genome editing and a loss of security and identity [37].

In Africa, there were two studies that assessed the perspective of human genome editing through the lens of Ubuntu, an African ethic, and from a group of researchers from the MENA region [37,42]. According to this perspective, the acceptance of human genome editing use as a therapeutic or management modality is predicated upon if the individual can attain personhood [36]. As such, if the individual were to lose autonomy because of its use, then therapeutic genome editing would be rejected as it would inhibit the ability to interact with the community leading to not attaining the level of a “fully actualized person.” [36]. Another study, which utilized an online discussion forum of 27 researchers from the healthcare sector in the MENA region, assessed their perspectives on the ethical implications of human genome editing [41]. Overall, there was a consensus that human gene editing could be a key treatment modality for genetic conditions; however, the concerns revolved around justice, discrimination, and harm [41]. Ultimately, it was found that while it could be a promising step in medical treatment and management, more research should be conducted, and potential regulations should be discussed [41].

In Asia, a study focused on Japanese perspectives of therapeutic human genomic editing in response to how the media has portrayed its use, particularly of the genome-edited twins in China carried out in 2018 [38]. Surveys were sent in 2016, 2018, and 2019 of which 3,100, 1,240, and 1,543 responded, respectively [38]. The surveys assessed acceptance of human genome editing if the media covered it for medical use and attempted to identify what factors of media coverage led to a negative impression [38]. Overall, when the media covered medical applications, there was a significantly favorable impression in both 2018 and 2019 (p=0.01) [38]. However, it was noted that the favorable impression trended downward to 13.5% in 2019 from 26.2% in 2018; this suggests that the news from China, and how the media portrayed it, were potentially the cause of the negative impact [38]. The leading parameter that caused the most dissent for genome editing was when the media covered the potential risks associated with it (p=0.01) [38]. Overall, when the news broke out regarding genome-edited twins in China, it appears that the Japanese still find human genome editing as a potential candidate for medical treatment but are still weary of the potential risks and harms associated with it [38].

In the Oceanic region, one study was conducted to assess the perspectives of human genomic editing in Aotearoa, New Zealand [39]. One such ethnic group, the Māori, has expressed strong opposition to modifying and editing organisms in the past [39]. The biggest issues and concerns that they had included the long-term effects, the potential cascade effect on future generations, and the effect on “mauri” - the Māori word for “life essence” [39]. The belief is that an individual’s “mauri” becomes altered or even destroyed when genome editing is incorporated [39]. There was an overall negative outlook toward human genome editing because of the risks and imbalance it brings to nature as a whole, including its use as a therapeutic modality [39].

There is also the perspective of future generations that must be taken into consideration in any discussion of human genetic engineering. In a biological sense, it is argued that genetic engineering could limit the heterozygosity of these future generations and that care must be taken to not confer any advantage in the children that are engineered and to limit any health risks that may come with the technology [43]. But with the worry of future generations being affected also comes the hope that prevents inherited diseases from being passed down [43]. In one study, sickle cell disease patients were found to be excited about the use of human genome editing if it meant that the disease would not be passed down to their children [42]. Many sickle cell patients and stakeholders indicated that they probably or would use genomic editing to give their babies a significantly reduced risk of serious disease over the course of their lifetime when compared to the general population [42].

Research Ethics

Preceding the parturition of gene-edited twins in China, Dr. He Jiankui issued ethical principles to “clarify for the public the clinical future of early-in-life genetic surgeries” or heritable genome editing [44]. He desired to repeat the naturally occurring genetic polymorphism in CCR5 that reduces HIV transmission in homozygous individuals [44]. The five principles are “mercy for families in need,” “only for serious disease-never vanity,” “respect a child’s autonomy,” “genes do not define you,” and “everyone deserves freedom from genetic disease” [10].

Mercy implies the relief of distress in the procedures performed, if not preventing it in the first place [10]. It was for this reason that Dr. He’s experimentations in gene editing were so decried, directly opposing this concept of mercy with the increased risk of infection that arose from his procedures [10]. Another important principle in genetic editing is that it should be used for disease, avoiding use for vanity [10]. Another problem with Dr. He’s editing of CCR5 is the risk to human diversity that may come with it [10].

The respect for children’s future autonomy is threatened by side effects of gene editing, such as susceptibility to the influenza virus caused by the ∆32 CCR5 mutations [10]. Regarding the principle of genes not defining the individual, the shared model of debility entails disabilities are not a function of deficiencies but are given by disabling barriers [10]. Finally, the principle holding that “everyone deserves freedom from genetic disease” emphasizes imbalances in health treatments, as not everyone is able to afford the same treatment [10].

In the context of human gene editing, the vulnerability clause applies when one party to a contract lacks decent alternatives to transacting on the other’s terms [44]. Adoption is one alternative, but not obtainable by everyone [44]. Finally, human gene editing used for therapeutic medical purposes will significantly improve people’s innate abilities. However, if this editing is only enjoyed by a few people, it will cause social disruption harming social equality and righteousness [45].

Substantiation suggests that women in germline editing trials are at amplified risk for obstetric problems such as placenta previa, abruption, and vasa previa [46]. Thus, germline editing trials must gauge the health outcomes of subjects to enable ethical, legal, and social implications [46]. Women moreover can be unreasonably harmed if there are no clearly set endpoints for trials [46]. Following the autonomy principle, there is also a need to ensure women’s consent to participate in trials [46].

Limitations of Included Studies

First, limited translational resources restricted the selected articles to only ones published in English. Although the inclusion criteria allowed for articles on ethics published worldwide, the language exclusion criteria limited the selection of articles to primary sources only. Second, other potential limitations include the short duration of the studies and the variety of ethical analyses that can vary depending on the year it was published.

Limitations of the Review Process

One limitation of the review process was that the search was limited to articles published only on or after 2013, not allowing for additional published data on ethics and genomic editing. Additionally, this review only included articles in English and in primary data formats such as original research articles and surveys, which may not represent all the articles on ethics and genomic editing. Finally, relevant articles may have been excluded due to searching only three databases combined with strict inclusion and exclusion criteria.

Future Research Considerations

While this scoping review provides a somewhat comprehensive assessment of the ethical implications of genomic editing from different vantage points, an analysis of government regulation may provide great benefit. Since genomic editing could potentially be used beyond in utero, further research regarding the ethical implication of its use for organoids, in vitro fertilization, stem cell therapy, human cloning, or sex selection would help understand what would be deemed ethically permissible. Finally, it would be noteworthy to assess current political stances and policy-making practices that have been created for genomic editing and determine how ethics play a role in their genesis.

## Conclusions

The ethics of human genome editing varies widely depending on the context. In philosophy, any issues that arise are outweighed by the medical benefit of disease eradication that is promised by genome editing, despite the extensive arguments centered around human dignity, the non-identity problem, and Habermas’ argument. Theological viewpoints all concur that therapeutic human genome editing does not violate ethical standards or human dignity, but more research and regulation are needed to prevent the technology from being misused. Public perspectives from the USA, Europe, Africa, and the MENA region all support therapeutic human genome editing when the medical benefits are highlighted, especially when there are fewer adverse effects on mom and fetus. And lastly, research ethics calls for more regulations in place for informed consent from female participants, ensuring autonomy for future children, and preventing further risk for future generations from inherited edits. Therefore, it can be concluded that therapeutic human genome editing has more support from the general population when given accurate risks and benefits of the technology, but further research and regulation are needed to ensure a safe and practical application. Furthermore, this scoping review is germane to the use of human genomic engineering from a “social” standpoint such as the genetic optimization of otherwise healthy children.

## Appendices

**Table 2:** The detailed search queries per database. Ab: abstract; Ti: title; Kw: keywords; Py: publication year; MP: Multi-purpose; TS: topic

S.N.	Embase	Results	Web of Science	Results	Ovid MEDLINE	Results
1	(“Genetic engineering”/exp) OR (“genetic manipulation”/exp)	164,848	TS = ("gen* edit*" OR "gen* engineer*" OR "gen* manipulation")	67,710	Exp Genetic Engineering/	223,931
2	(“Gen* edit*”: ab, ti, kw) OR (“gen* engineer*”: ab, ti, kw) OR (“gen* manipulation”: ab, ti, kw)	68,495	TS = ("clustered regularly interspaced short palindromic repeats" OR crispr* OR meganuclease* OR "zinc finger nuclease" OR "transcription activator like effector nuclease*" OR talen)	35,685	("Gen* edit*" OR "gen* engineer*" OR "gen* manipulation"). Mp.	75,226
3	(“Clustered regularly interspaced short palindromic repeat”/exp) OR (“crispr cas system”/exp)	46,599	TS = (ethic* OR moral* OR bioethic*)	425,596	Exp Clustered Regularly Interspaced Short Palindromic Repeats/	4,975
4	(“Clustered regularly interspaced short palindromic repeats”: ab, ti, kw) OR (crispr*: ab, ti, kw) OR (meganuclease*: ab, ti, kw) OR (“zinc finger nuclease”: ab, ti, kw) OR (“transcription activator like effector nuclease*”: ab, ti, kw) OR (talen: ab, ti, kw)	46,492	(TS = {"gen* edit*" OR "gen* engineer*" OR "gen* manipulation"}) OR (TS = {"clustered regularly interspaced short palindromic repeats" OR crispr* OR meganuclease* OR "zinc finger nuclease" OR "transcription activator like effector nuclease*" OR talen})	90,734	Exp CRISPR-Cas Systems/	15,161
5	(“Ethics”/exp) OR (“morality”/exp)	363,185	({TS = ("gen* edit*" or "gen* engineer*" or "gen* manipulation")} OR {TS = ("clustered regularly interspaced short palindromic repeats" OR crispr* OR meganuclease* OR "zinc finger nuclease" OR "transcription activator like effector nuclease*" OR talen)}) AND (TS = {ethic* OR moral* OR bioethic*})	1,635	Exp Ethics/	153,766
6	(Ethic*: ab, ti, kw) OR (moral*: ab, ti, kw) OR (bioethic*: ab, ti, kw)	265,004	({TS = ("gen* edit*" or "gen* engineer*" or "gen* manipulation")} OR {TS = ("clustered regularly interspaced short palindromic repeats" OR crispr* OR meganuclease* OR "zinc finger nuclease" OR "transcription activator like effector nuclease*" OR talen)}) AND (TS = {ethic* OR moral* OR bioethic*}) from 2013-2022	1,157	Exp Morals/	179,597
7	(“Animal”/exp OR “animal experiment”/exp OR “animal model”/exp OR “invertebrate”/exp OR “animal tissue”/exp OR “animal cell”/exp OR “nonhuman”/exp) NOT (“human”/exp OR “normal human”/exp)	7,639,438	-	-	(Ethic* OR moral* OR bioethic*). Mp.	273,195
8	(“Genetic engineering”/exp OR “genetic manipulation”/exp) OR (“gen* edit*”: ab, ti, kw OR “gen* engineer*”: ab, ti, kw OR “gen* manipulation”: ab, ti, kw) OR (“clustered regularly interspaced short palindromic repeat”/exp OR “crispr cas system”/exp) OR (“clustered regularly interspaced short palindromic repeats”: ab, ti, kw OR crispr*: ab, ti, kw OR meganuclease*: ab, ti, kw OR “zinc finger nuclease”: ab, ti, kw OR “transcription activator like effector nuclease*”: ab, ti, kw OR talen: ab, ti, kw)	215,899	-	-	("Clustered regularly interspaced short palindromic repeats" OR crispr* OR meganuclease* OR "zinc finger nuclease" OR "transcription activator like effector nuclease*" OR talen). Mp.	36,007
9	(“Ethics”/exp OR “morality”/exp) OR (ethic*: ab, ti, kw OR moral*: ab, ti, kw OR bioethic*: ab, ti, kw)	482,370	-	-	(Exp Genetic Engineering/) OR ({"Gen* edit*" OR "gen* engineer*" OR "gen* manipulation"}. Mp.) OR (Exp Clustered Regularly Interspaced Short Palindromic Repeats/) OR (Exp CRISPR-Cas Systems/) OR ({"Clustered regularly interspaced short palindromic repeats" OR crispr* OR meganuclease* OR "zinc finger nuclease" OR "transcription activator like effector nuclease*" OR talen}. Mp.)	278,768
10	({“Genetic engineering”/exp OR “genetic manipulation”/exp} OR {“gen* edit*”: ab, ti, kw OR “gen* engineer*”: ab, ti, kw OR “gen* manipulation”: ab, ti, kw} OR {“clustered regularly interspaced short palindromic repeat”/exp OR “crispr cas system”/exp} OR {“clustered regularly interspaced short palindromic repeats”: ab, ti, kw OR crispr*: ab, ti, kw OR meganuclease*: ab, ti, kw OR “zinc finger nuclease”: ab, ti, kw OR “transcription activator like effector nuclease*”: ab, ti, kw OR talen: ab, ti, kw}) AND ({“ethics”/exp OR “morality”/exp} OR {ethic*: ab, ti, kw OR moral*: ab, ti, kw OR bioethic*: ab, ti, kw})	4,233	-	-	(Exp Ethics/) OR (Exp Morals/) OR ({Ethic* OR moral* OR bioethic*}. Mp.)	320,345
11	({“Genetic engineering”/exp OR “genetic manipulation”/exp} OR {“gen* edit*”: ab, ti, kw OR “gen* engineer*”: ab, ti, kw OR “gen* manipulation”: ab, ti, kw} OR {“clustered regularly interspaced short palindromic repeat”/exp OR “crispr cas system”/exp} OR {“clustered regularly interspaced short palindromic repeats”: ab, ti, kw OR crispr*: ab, ti, kw OR meganuclease*: ab, ti, kw OR “zinc finger nuclease”: ab, ti, kw OR “transcription activator like effector nuclease*”: ab, ti, kw OR talen: ab, ti, kw}) AND ({“ethics”/exp OR “morality”/exp} OR {ethic*: ab, ti, kw OR moral*: ab, ti, kw OR bioethic*: ab, ti, kw}) AND [2013-2022]/py	1,897	-	-	({Exp Genetic Engineering/} OR {"Gen* edit*" OR "gen* engineer*" OR "gen* manipulation"}. Mp.) OR (Exp Clustered Regularly Interspaced Short Palindromic Repeats/) OR (Exp CRISPR-Cas Systems/) OR ({"Clustered regularly interspaced short palindromic repeats" OR crispr* OR meganuclease* OR "zinc finger nuclease" OR "transcription activator like effector nuclease*" OR talen}. Mp.) AND ({Exp Ethics/} OR (Exp Morals/) OR ({Ethic* OR moral* OR bioethic*}. Mp.})	4,791
12	({“genetic engineering”/exp OR “genetic manipulation”/exp} OR (“gen* edit*”: ab, ti, kw OR “gen* engineer*”: ab, ti, kw OR “gen* manipulation”: ab, ti, kw) OR (“clustered regularly interspaced short palindromic repeat”/exp OR “crispr cas system”/exp) OR (“clustered regularly interspaced short palindromic repeats”: ab, ti, kw OR crispr*: ab, ti, kw OR meganuclease*: ab, ti, kw OR “zinc finger nuclease”: ab, ti, kw OR “transcription activator like effector nuclease*”: ab, ti, kw OR talen: ab, ti, kw)} AND {(“ethics”/exp OR “morality”/exp) OR (ethic*: ab, ti, kw OR moral*: ab, ti, kw OR bioethic*: ab, ti, kw}) AND ([2013-2022]/py) NOT ({“animal”/exp OR “animal experiment”/exp OR “animal model”/exp OR “invertebrate”/exp OR “animal tissue”/exp OR “animal cell”/exp OR “nonhuman”/exp) NOT (“human”/exp OR “normal human”/exp})	1,774	-	-	Limit ({(Exp Genetic Engineering/) OR {("Gen* edit*" OR "gen* engineer*" OR "gen* manipulation"). Mp.} OR (Exp Clustered Regularly Interspaced Short Palindromic Repeats/) OR (Exp CRISPR-Cas Systems/) OR {("Clustered regularly interspaced short palindromic repeats" OR crispr* OR meganuclease* OR "zinc finger nuclease" OR "transcription activator like effector nuclease*" OR talen). Mp.}} AND {(Exp Ethics/) OR (Exp Morals/) OR {(Ethic* OR moral* OR bioethic*). Mp.}}) to yr = “2013-2022”	1,580
13	-	-	-	-	(Limit ({(Exp Genetic Engineering/) OR {("Gen* edit*" OR "gen* engineer*" OR "gen* manipulation"). Mp.} OR (Exp Clustered Regularly Interspaced Short Palindromic Repeats/) OR (Exp CRISPR-Cas Systems/) OR {("Clustered regularly interspaced short palindromic repeats" OR crispr* OR meganuclease* OR "zinc finger nuclease" OR "transcription activator like effector nuclease*" OR talen). Mp.}} AND {(Exp Ethics/) OR (Exp Morals/) OR {(Ethic* OR moral* OR bioethic*). Mp.}}) to (yr = “2013-2022”) NOT (Animals/ not {Animals/ and Humans/})	1,514

## References

[1] Barman A, Deb B, Chakraborty S (2020). A glance at genome editing with CRISPR-Cas9 technology. Curr Genet.

[2] (2022). CRISPR bombshell: Chinese researcher claims to have created gene-edited twins. https://www.science.org/content/article/crispr-bombshell-chinese-researcher-claims-have-created-gene-edited-twins.

[3] Zhou Q, Zhang Y, Zou Y, Yin T, Yang J (2020). Human embryo gene editing: God's scalpel or Pandora's box?. Brief Funct Genomics.

[4] Akram F, Sahreen S, Aamir F (2022). An insight into modern targeted genome-editing technologies with a special focus on CRISPR/Cas9 and its applications. Mol Biotechnol.

[5] Asmamaw M, Zawdie B (2021). Mechanism and applications of CRISPR/Cas-9-mediated genome editing. Biologics.

[6] Cribbs AP, Perera SM (2017). Science and bioethics of CRISPR-Cas9 gene editing: an analysis towards separating facts
and fiction. Yale J Biol Med.

[7] Golkar Z (2020). CRISPR: a journey of gene-editing based medicine. Genes Genomics.

[8] El Ouar I, Djekoun A (2021). Therapeutic and diagnostic relevance of CRISPR technology. Biomed Pharmacother.

[9] Morar N (2015). An empirically informed critique of Habermas' argument from human nature. Sci Eng Ethics.

[10] Petre I (2017). Future generations and the justifiability of germline engineering. J Med Philos.

[11] Chen AA, Zhang X (2022). Rethinking the knowledge-attitudes model and introducing belief in human evolution: examining antecedents of public acceptability of human gene editing. Health, Risk & Society.

[12] Dabrock P (2009). Playing God? Synthetic biology as a theological and ethical challenge. Syst Synth Biol.

[13] Shinwari ZK, Tanveer F, Khalil AT (2018). Ethical issues regarding CRISPR mediated genome editing. Curr Issues Mol Biol.

[14] Ayanoğlu FB, Elçin AE, Elçin YM (2020). Bioethical issues in genome editing by CRISPR-Cas9 technology. Turk J Biol.

[15] Pepper MS, Pope A, Kling S, Alessandrini M, Van Staden W, Green RJ (2018). Ethical considerations in the application of cell and gene therapies in children. S Afr Med J.

[16] Kang X, He W, Huang Y (2016). Introducing precise genetic modifications into human 3PN embryos by CRISPR/Cas-mediated genome editing. J Assist Reprod Genet.

[17] Plaza Reyes A, Lanner F (2017). Towards a CRISPR view of early human development: applications, limitations and ethical concerns of genome editing in human embryos. Development.

[18] Schleidgen S, Dederer HG, Sgodda S, Cravcisin S, Lüneburg L, Cantz T, Heinemann T (2020). Human germline editing in the era of CRISPR-Cas: risk and uncertainty, inter-generational responsibility, therapeutic legitimacy. BMC Med Ethics.

[19] Almeida M, Diogo R (2019). Human enhancement: genetic engineering and evolution. Evol Med Public Health.

[20] Daniel VM (2013). Genomics and Genetic Engineering: Playing God?. https://www.igi-global.com/chapter/genomics-genetic-engineering/76066.

[21] Aiyegbusi OL, Macpherson K, Elston L (2020). Patient and public perspectives on cell and gene therapies: a systematic review. Nat Commun.

[22] Segers S, Mertes H (2020). Does human genome editing reinforce or violate human dignity?. Bioethics.

[23] Chan DK (2015). The concept of human dignity in the ethics of genetic research. Bioethics.

[24] Raposo VL (2019). Gene editing, the mystic threat to human dignity. J Bioeth Inq.

[25] Alonso M, Savulescu J (2021). He Jiankui´s gene-editing experiment and the non-identity problem. Bioethics.

[26] Li J, Zhang X (2019). Should parents design their children's genome: some general arguments and a Confucian solution. Philosophies.

[27] Gyngell C, Douglas T, Savulescu J (2017). The ethics of germline gene editing. J Appl Philos.

[28] Alsomali N, Hussein G (2021). CRISPR-Cas9 and He Jiankui's case: an Islamic bioethics review using Maqasid al-Shari'a and Qawaid Fighiyyah. Asian Bioeth Rev.

[29] Isa NM, Zulkifli NA, Man S (2020). Islamic perspectives on CRISPR/Cas9-mediated human germline gene editing: a preliminary discussion. Sci Eng Ethics.

[30] Loike JD, Kadish A (2018). Outer limits of biotechnologies: a Jewish perspective. Rambam Maimonides Med J.

[31] Peters T (2017). Should CRISPR scientists play God?. Religions.

[32] Peters T (2019). Flashing the yellow traffic light: choices forced upon us by gene editing technologies. [Article in Korean]. Theol Sci.

[33] Rubeis G, Steger F (2018). Risks and benefits of human germline genome editing: an ethical analysis. Asian Bioeth Rev.

[34] Vasquez-Loarte TC, Lucas TL, Harris-Wai J, Bowen DJ (2020). Beliefs and values about gene therapy and in-utero gene editing in patients with hemophilia and their relatives. Patient.

[35] Howell EL, Kohl P, Scheufele DA, Clifford S, Shao A, Xenos MA, Brossard D (2022). Enhanced threat or therapeutic benefit? Risk and benefit perceptions of human gene editing by purpose and heritability of edits. J Risk Res.

[36] Shozi B (2021). Does human germline genome editing violate human dignity? An African perspective. J Law Biosci.

[37] Ebeling S, Gebhard U (2022). Ideas, hopes, and fears: what young adults think about genome editing, nature, and society. Cult Stud of Sci Educ.

[38] Watanabe D, Saito Y, Tsuda M, Ohsawa R (2020). Increased awareness and decreased acceptance of genome-editing technology: the impact of the Chinese twin babies. PLoS One.

[39] Hudson M, Mead AT, Chagné D, Roskruge N, Morrison S, Wilcox PL, Allan AC (2019). Indigenous perspectives and gene editing in Aotearoa New Zealand. Front Bioeng Biotechnol.

[40] Hendriks S, Giesbertz NA, Bredenoord AL, Repping S (2018). Reasons for being in favour of or against genome modification: a survey of the Dutch general public. Hum Reprod Open.

[41] Abuhammad S, Khabour OF, Alzoubi KH (2021). Researchers views about perceived harms and benefits of gene editing: a study from the MENA region. Heliyon.

[42] Hollister BM, Gatter MC, Abdallah KE (2019). Perspectives of sickle cell disease stakeholders on heritable genome editing. CRISPR J.

[43] Getz LJ, Dellaire G (2020). Back to basics: application of the principles of bioethics to heritable genome interventions. Sci Eng Ethics.

[44] Malmqvist E (2021). Clinical trials of germline gene editing: the exploitation problem. Bioethics.

[45] Zang S, Yueqin C (2021). How the gene-editing in medicine and public health practice could stand the test of bioethics. Acta Bioethica.

[46] Farrell RM, Michie M, Scott CT, Flyckt R, LaPlante M (2019). Prioritizing women's health in germline editing research. AMA J Ethics.

